# ROENet: A ResNet-Based Output Ensemble for Malaria Parasite Classification

**DOI:** 10.3390/electronics11132040

**Published:** 2022-06-29

**Authors:** Ziquan Zhu, ShuiHua Wang, YuDong Zhang

**Affiliations:** 1 School of Computing and Mathematical Sciences, University of Leicester, East Midlands, Leicester, LE1 7RH, UK

**Keywords:** malaria, randomized neural network, ResNet-18, convolutional neural network, output ensemble, blood smear

## Abstract

**Background:**

People may be infected with an insect-borne disease (malaria) through the blood input of malaria-infected people or the bite of Anopheles mosquitoes. Doctors need a lot of time and energy to diagnose malaria, and sometimes the results are not ideal. Many researchers use CNN to classify malaria images. However, we believe that the classification performance of malaria parasites can be improved.

**Methods:**

In this paper, we propose a novel method (ROENet) to automatically classify malaria parasite on the blood smear. The backbone of ROENet is the pretrained ResNet-18. We use randomized neural networks (RNNs) as the classifier in our proposed model. Three RNNs are used in ROENet, which are random vector functional link (RVFL), Schmidt neural network (SNN), and extreme learning machine (ELM). To improve the performance of ROENet, the results of ROENet are the ensemble outputs from three RNNs.

**Results:**

We evaluate the proposed ROENet by five-fold cross-validation. The specificity, F1 score, sensitivity, and accuracy are 96.68 ± 3.81%, 95.69 ± 2.65%, 94.79 ± 3.71%, and 95.73 ± 2.63%, respectively.

**Conclusions:**

The proposed ROENet is compared with other state-of-the-art methods and provides the best results of these methods.

## 1 Introduction

People may be infected with an insect-borne disease (malaria) through the blood input of malaria-infected people or the bite of Anopheles mosquitoes. Colds, periodic and regular attacks, fever, and sweating are several characteristics of patients infected with malaria. After many attacks for a long time, it can cause anemia and splenomegaly. Thus far, the harmful impact of malaria in the world is still very serious. People living in malaria-endemic areas account for about 40% of the total human population. Malaria is one of the most feared diseases for people living on the African continent. Every year, malaria patients on the African continent account for 90% of malaria patients in the world. Malaria is the cause of death of more than two million people worldwide every year. Malaria is also prevalent in Southeast and Central Asia. The infection sources of malaria are roughly divided into two categories: (i) malaria patients and (ii) asymptomatic carriers containing gametophytes. The transmission probability of malaria increases with the increase in gametophyte density.

The earlier malaria is diagnosed, the more conducive it is for treatment of patients. Thus far, the diagnostic methods of malaria in the hospital are as follows: (i) clinical manifestation, (ii) molecular biological technology diagnosis, and (iii) therapeutic diagnosis. However, these diagnostic methods require doctors to spend a long period of time when diagnosing. This long diagnosis process may delay the timely treatment of patients. Moreover, doctors are easily disturbed by some factors in the diagnosis process, such as lack ofsleep, illness, and so on. Therefore, the process of diagnosing malaria by doctors is very inefficient.

Many researchers are willing to apply computer technology to malaria diagnosis. Manescu et al. [1] introduced a new model (DeepMCNN), which was based on the convolution neural network. The introduced DeepMCNN obtained 0.92 sensitivity and 0.90 specificity. Yang et al. [2] introduced a novel method to detect malaria. This method combined IGMS and CNN and obtained 93.46 ± 0.32% accuracy, 92.59 ± 1.27% sensitivity, 94.33 ± 1.25% specificity, and 94.25 ± 1.13% precision. Shoohi and Saud [3] introduced a method for malaria image classification. This method (Deep Convolutional Generative Adversarial Network) was based on CNN. Mehanian et al. [4] used deep learning to classify malaria images. Mukherjee et al. [5] introduced a novel method to classify malaria images. The proposed method was based on the convolution neural network and obtained a 0.95 dice score. Khadim et al. [6] evaluated several different activation functions to detect malaria in the CNN model. These several different activation functions were Sigmoid, Tanh, Leaky ReLU, ReLU, and Swish. Based on the experimental results, the Swish activation function yielded better results than other activation functions. Magotra and Rohil [7] introduced a novel model (lightweight CNN) to detect malaria. Two classical models were used to compare with the lightweight CNN, which were VGG-19 and Inception-v3. This lightweight CNN model could obtain 96% accuracy. At the same time, this model can reduce training time and computations. Marques et al. [8] proposed a new model to detect malaria based on the CNN model. In this model, EfficientNet was used as the backbone model. This model achieved 97.74% precision, 99.76% ROC, 98.82% recall, and 98.28% F1. Sarkar et al. [9] presented a shallow-approach CNN model. The proposed model could reduce the run time. Raihan and Nahid [10] introduced an explainable CNN model to classify malaria. This explainable CNN model was composed of CNN, wavelet packet 2d, and Whale Optimization Algorithm. This model obtained 94.39% precision, 94.80% F1, 94.78% accuracy, and 95.21% recall.

From the above description of malaria diagnosis by computer technology [11], it can be concluded that most scholars use the CNN model for experiments. However, we believe that the classification performance of malaria parasite can be improved. We propose a new model (ROENet) to automatically classify malaria parasites on the blood smears. ROENet means that the model is a ResNet-based output ensemble for malaria parasite classification. The main contributions of this study are the following:

- A novel method (ROENet) is proposed to automatically classify malaria parasite on the blood smear.
- The fine-tuned ResNet-18 is the feature extraction.
- Three RNNs are selected to replace the last five layers of the fine-tuned ResNet-18.
- Three RNNs are selected as the classifier of the proposed ROENet.
- The final outputs of ROENet are the ensemble outputs from three RNNs.

The remainder of the study is organized as follows: the public dataset is demonstrated in Section 2; Section 3 details the method; the experiment settings and results are presented in Section 4; Section 5 is the conclusion.

## 2 Materials

**Figure 1:**
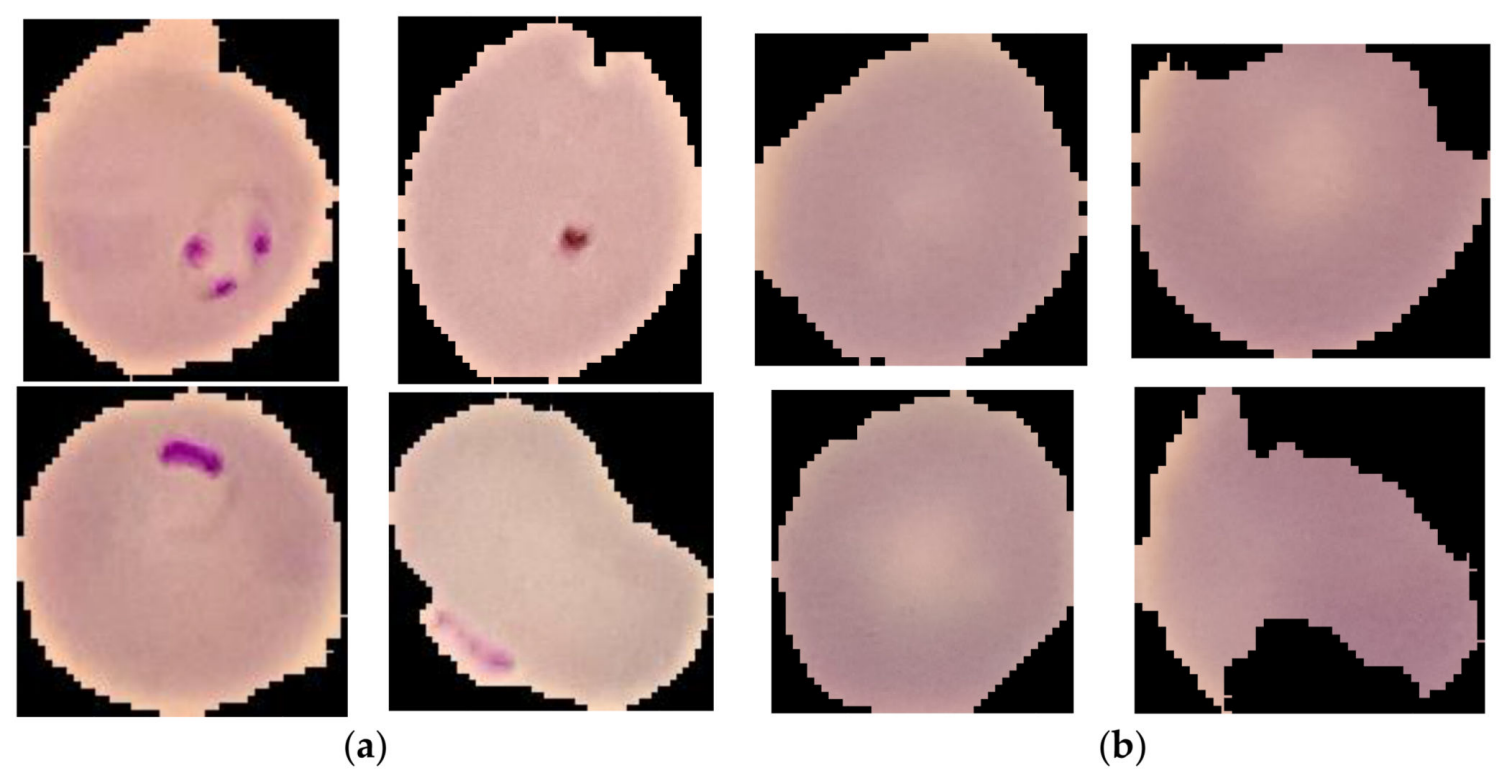
Some of the parasitized and uninfected images. (**a**) Parasitized images; (**b**) uninfected images.

The malaria images are available on the NIH website, which the Chittagong Medical College Hospital prepared. This dataset is public. Giemsa-stained thin blood smears were from 150 patients infected with Plasmodium falciparum and 50 healthy patients in Chittagong Medical College Hospital, Bangladesh. These stained thin blood smears were photographed. The slide image of each micro field of view was taken through the built-in camera in the smartphone. These images were manually marked by experts from Oxford Tropical Medicine Research Center in Mahilon, Bangkok, Thailand. There are 27,558 malaria images in total, including 13,779 images of parasitized and 13,779 images of uninfected. Some of the parasitized and uninfected images are provided in Figure 1. The image processing method is used in this open dataset to find parasites in the digital image of blood film. The typical shape, data, and visual appearance of parasites are marked manually by experts. If there is no expert mark, the image is uninfected.

## 3 Methods’ Results

### 3.1 Proposed ROENet

The acronym and full explanation table is provided in Table 1. As more and more scholars research image analysis, image analysis technology continues to progress [12]. One of the most significant steps in the analysis of the image is to extract features from images [13]. However, each image contains too much content. Extracting useful features in the image quickly and accurately has been perplexing to scholars. Previously, scholars manually extracted features from the image [14]. However, the process of manually extracting features was very time consuming, and the results were often not ideal. More and more scholars were applying computer technology to image analysis and proposed many CNN models [15], such as AlexNet, ResNet, etc. In the CNN model, the convolution layers and pooling layers can reduce the number of parameters. In this situation, calculation and experimental time are greatly reduced. This is one of the reasons why the CNN model is so popular [16].

**Figure 2:**
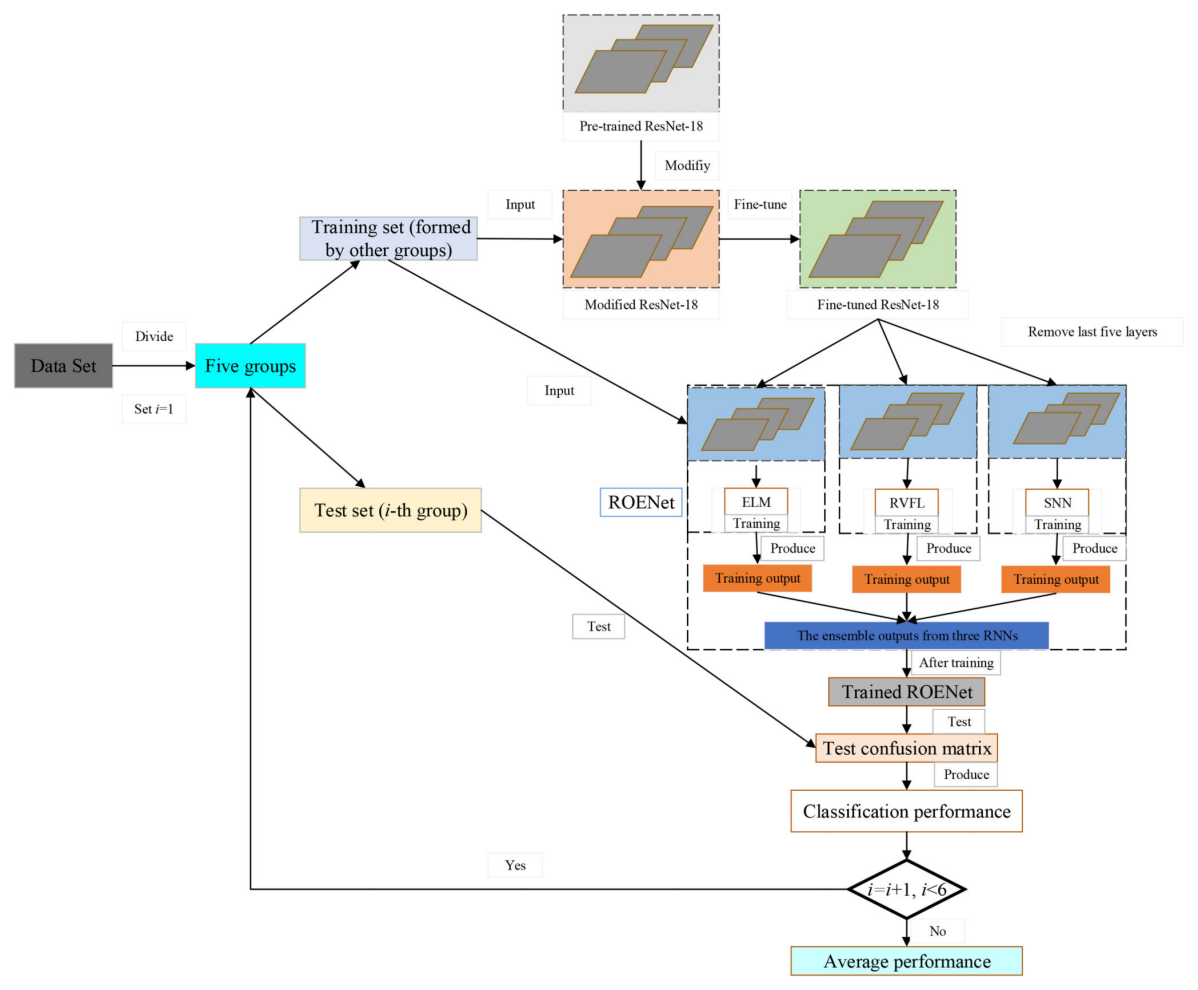
The flowchart of the proposed ROENet.

This paper proposes a novel method (ROENet) to automatically classify malaria parasite on the blood smear. The public dataset can be downloaded on the NIH website. The backbone of ROENet is the pre-trained ResNet-18. ResNet-18 was pre-trained on the ImageNet. Therefore, the output nodes of the pre-trained ResNet-18 are 1000. However, the output nodes are two in this paper. Therefore, we perform some modifications to the backbone. We chose randomized neural networks (RNNs) as the classifier in our proposed model. Three RNNs are used in ROENet, which include random vector functional link (RVFL) [17], Schmidt neural network (SNN) [18], and extreme learning machine (ELM) [19]. The results of ROENet are the ensemble outputs from three RNNs to improve the performance. Table 2 demonstrates the pseudocode of ROENet. The flowchart of ROENet is provided in Figure 2.

### 3.2 Backbone of ROENet

The depth of the network has a great influence on the performance of the CNN model. In theory, with the deepening of the network depth, the model should achieve better performance [20]. However, the performance of deeper networks is not necessarily good. When the depth of the CNN model continues to increase, the CNN model may encounter the degradation problem [21]. The performance of the model stagnates or even decreases when the number of layers of the CNN model increases. This is the problem of degradation [22].

The degradation problem at least shows that the network model is difficult to train. By adding new layers, the network model increases the depth continuously [23]. Sometimes, the newly added layers learn nothing and simply copy the features of the previous layer. This is identity mapping [24]. This can ensure that the performance of the network model will not degrade. Residual learning solves the degradation problem in this way [25]. For a CNN structure (formed by stacking several layers), *X* is the input, *P(X)* is the learned feature, and the feature extracted by residual learning is recorded as *Q(X)*.

(1) Q(X)=P(X)-X

(2) P(X)=Q(X)+X

**Figure 3:**
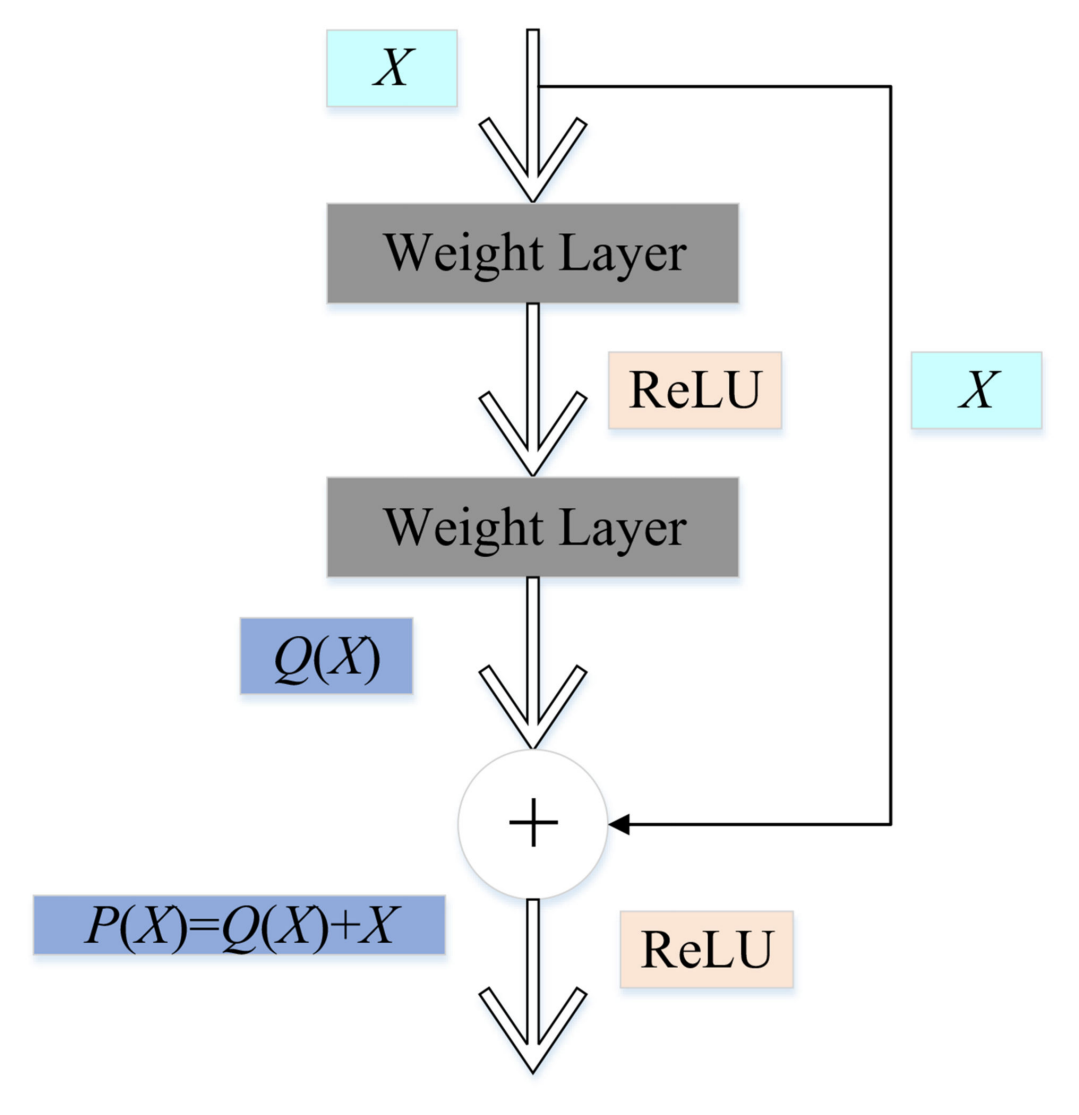
The structure of the residual learning.

It can be seen from the above formula that the worst case for residual learning is that the residual is 0, so the stacking layer just completes the identity mapping [26]. When the residual is not 0, the stacking layer will learn new features to improve the network’s performance. Therefore, residual learning can cope with the degradation problem [27]. The residual learning structure is demonstrated in Figure 3.

**Figure 4:**
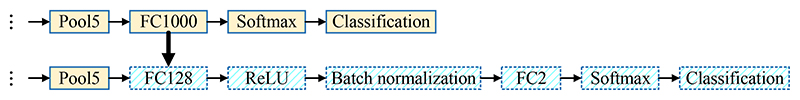
The modifications of the backbone.

The backbone of ROENet is the pre-trained ResNet-18. The output nodes of the pretrained ResNet-18 are 1000. However, the output nodes are two in this paper. Therefore, we perform some modifications to the backbone. The modifications of the backbone of the proposed ROENet are provided in Figure 4. FC1000, softmax, and the classification layer are removed. We add FC128, ReLU, BN, FC2, softmax, and the classification layer.

### 3.3 Classifier of ROENet

**Figure 5:**
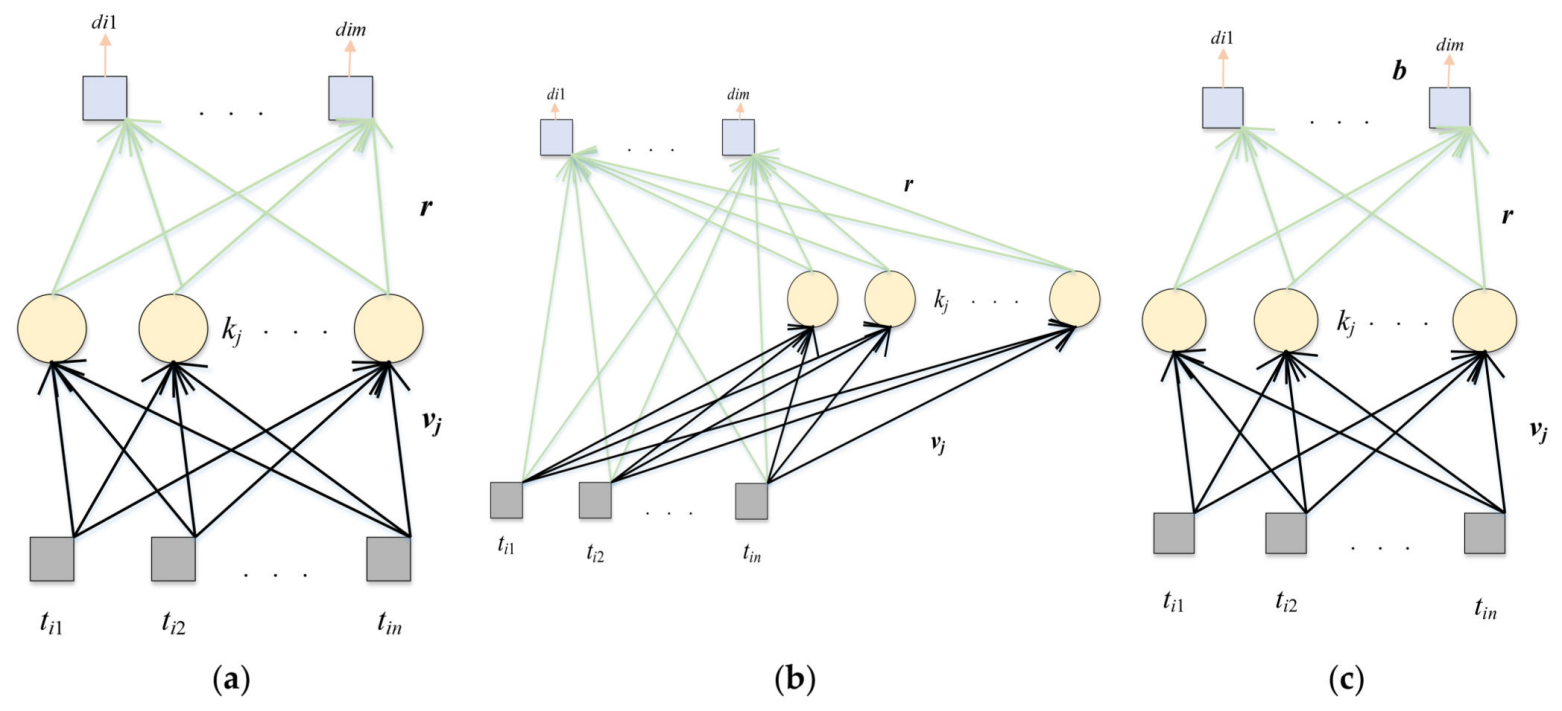
The structures of three RNNs. (**a**) ELM; (**b**) RVFL; (**c**) SNN.

There are many layers in the CNN model, and each layer has many parameters. The randomized neural networks (RNNs) have only three simple layers: input layer, hidden layer, and output layer. Only the shallow structure of the three-layer RNN model can effectively alleviate the overfitting problem. The parameters (the randomized weights and biases) in the RNN model are also trained quickly. Because RNN has good classification performance, it has been applied to many machine learning tasks, such as geography, big data analysis, chemistry, and so on. Three RNNs are used in this paper, which are ELM, RVFL, and SNN. ELM projects the input features into the hidden space randomly and does not need gradient-based backpropagation to adjust the weights [28]. The most obvious structural difference between RVFL and ELM is that there is a quick connection between input and output in RVFL [29]. This quick connection can effectively improve the classification performance of RVFL and the robustness of the model. SNN [30] was an RNN model proposed by Schmidt, Kraijveld, and Duin 30 years ago. The structure of SNN is consistent with that of ELM. However, in the SNN model, the output layer has a learnable output bias. These three RNN models are very classical and have achieved excellent classification performance since they were proposed. Their structures are provided in Figure 5.

As can be seen from the above figure, the structures of these three RNNs used in this paper are different. RNNs have only a three-layer structure; thus, the calculation method is almost identical. Suppose there is a dataset (***t**_i_, **y**_i_*) and the dataset contains *i*-th sample:

(3) $$t_{i}={\left(t_{i1},\ldots ,t_{in}\right)}^{\text{T}}\in {\text{R}}^{n},i=1,\ldots ,N,$$

(4) $$y_{i}={\left(y_{i1},\ldots ,y_{im}\right)}^{\text{T}}\in {\text{R}}^{m},i=1,\ldots ,N,$$

where *N* is the number of different samples, the input dimension is represented by *n*, and *m* is the output dimension.

For ELM, we chave the following:

(5) $$M_{ELM(i)}=\sum_{j=1}^{u}l\left(v_{j}t_{i}+K_{j}\right),i=1,\ldots ,N$$

where *V_j_* represents the weight from the input node to the *j*-th node in the hidden layer, *K_j_* represents the bias of the *j*-th node in the hidden layer, the sigmoid function is demonstrated by *I*, and *u* is the number of hidden nodes in the hidden layer.

For RVFL, this calculation is a step further:

(6) $$M_{RVFL(i)}=\;\text{concat}(T,E)$$

where the input matrix is **T** = ***(t_t_*,…, *t_N_)^T^***.

(7) $$E_{RVFL(i)}=\sum_{j=1}^{u}l\left(v_{j}t_{i}+K_{j}\right),i=1,\ldots ,N$$

For SNN, we have the following.

(8) $${M\text{SNN}}_{\left(i\right)}=\sum_{j=1}^{u}l\left(v_{j}t_{i}+K_{j}\right),i=1,\ldots ,N$$

For ELM and RVFL, the final output weights are calculated as follows:

(9) $$r=M_{net}^{+}Y$$

where **r** is the final output weight, the pseudo-inverse matrix of **M_net_** is $M_{\text{net}}^{+}$, and the ground-truth label of the dataset is **Y = (*y*_1_,…,*y_N_*)^T^**.

For SNN, there are biases (**b**) between the hidden layer and output layer:

(10) $$(r,b)=M_{\text{net}}^{+}Y$$

where $M_{\text{net}}^{+}$ is the pseudo-inverse matrix of $\left(\begin{array}{l} M_{\text{net}}^{+}\\ 1 \end{array}\right)$.

Although the RNN model is simple, bad weights and biases will seriously affect the classification performance. Therefore, in this paper, we combine the results of three RNN models to obtain the final classification model based on majority voting. Because the three RNN models used in this paper have some differences, it is more helpful to obtain diversified information in order to further improve the performance and robustness of the system.

### 3.4 Evaluation

The parasitized images are defined as the positive, and the uninfected images are defined as the negative. We evaluate the proposed ROENet by five-fold cross-validation. Five measures are selected, which are sensitivity (Se), accuracy (Ac), F1 score (F1), and specificity (Sp).

(11) $$\{\begin{array}{l} \text{Se}=\frac{\text{TP}}{\text{TP}+\text{FN}}\\ \text{Ac}=\frac{\text{TP}+\text{TN}}{\text{TP}+\text{TN}+\text{FP}+\text{FN}}\\ \text{F}1=\frac{2\times \text{TP}}{2\text{TP}+\text{FP}+\text{FN}}\\ \text{Sp}=\frac{\text{TN}}{\text{TN}+\text{FP}} \end{array},$$

## 4 Experiment Settings and Results

### 4.1 Experiment Settings

We set the max-epoch to 4 to prevent the overfitting problem. The learning rate is set as **10**^-4^. The minibatch size is 128. The dataset used in this paper is small and the batch size is large; thus, the convergence is fast. The number of the hidden nodes (***u***) in the hidden layer is 400. Table 3 provides the hyper-parameters of the proposed ROENet.

### 4.2 The Performance of ROENet

The classification performance of ROENet is provided in Table 4. In this paper, we evaluate the proposed ROENet by five-fold cross-validation. The F1 score (F1), specificity (Sp), accuracy (Ac), and sensitivity (Se) are 95.69 ± 2.65%, 96.68 ± 3.81%, 95.73 ± 2.63%, and 94.79 ± 3.71%, respectively. The average values of all results are greater than 94%. These results prove that the model in this paper is a good choice to classify malaria parasite on the blood smear.

### 4.3 Comparison of Different Backbones

**Figure 6:**
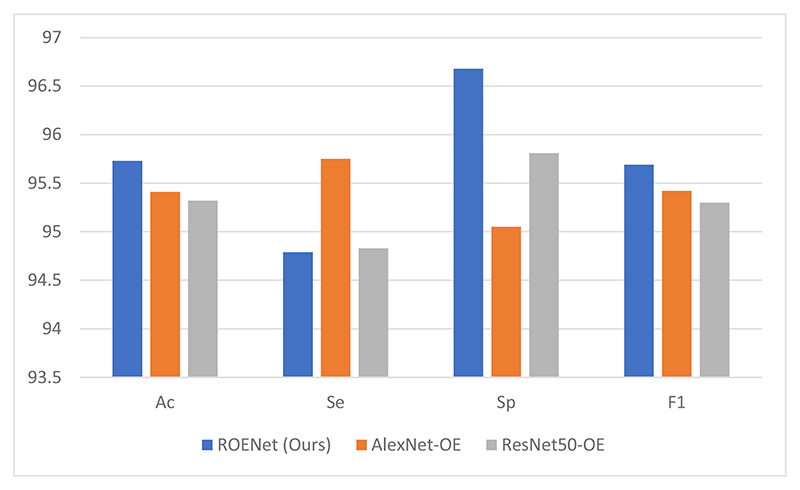
The comparison of different backbones.

We test different backbones, which are AlexNet and ResNet-50. The classification performances of these different backbones are presented in Table 4. The different backbones comparison figure is shown in Figure 6. Because ResNet-18 can achieve the best classification results when it is used as the backbone model based on the experimental results, ResNet-18 is selected as the preferred architecture in this paper.

Our model achieves the best results in accuracy, specificity, and F1 score in comparison to the other two models. AlexNet contains too many parameters, which could cause degradation problems. ResNet-50 has more layers than ResNet-18. Therefore, ResNet-50 may be more likely to meet gradient vanishing problems. Therefore, our model obtains better results than other models.

### 4.4 Effects of Output Ensemble

**Figure 7:**
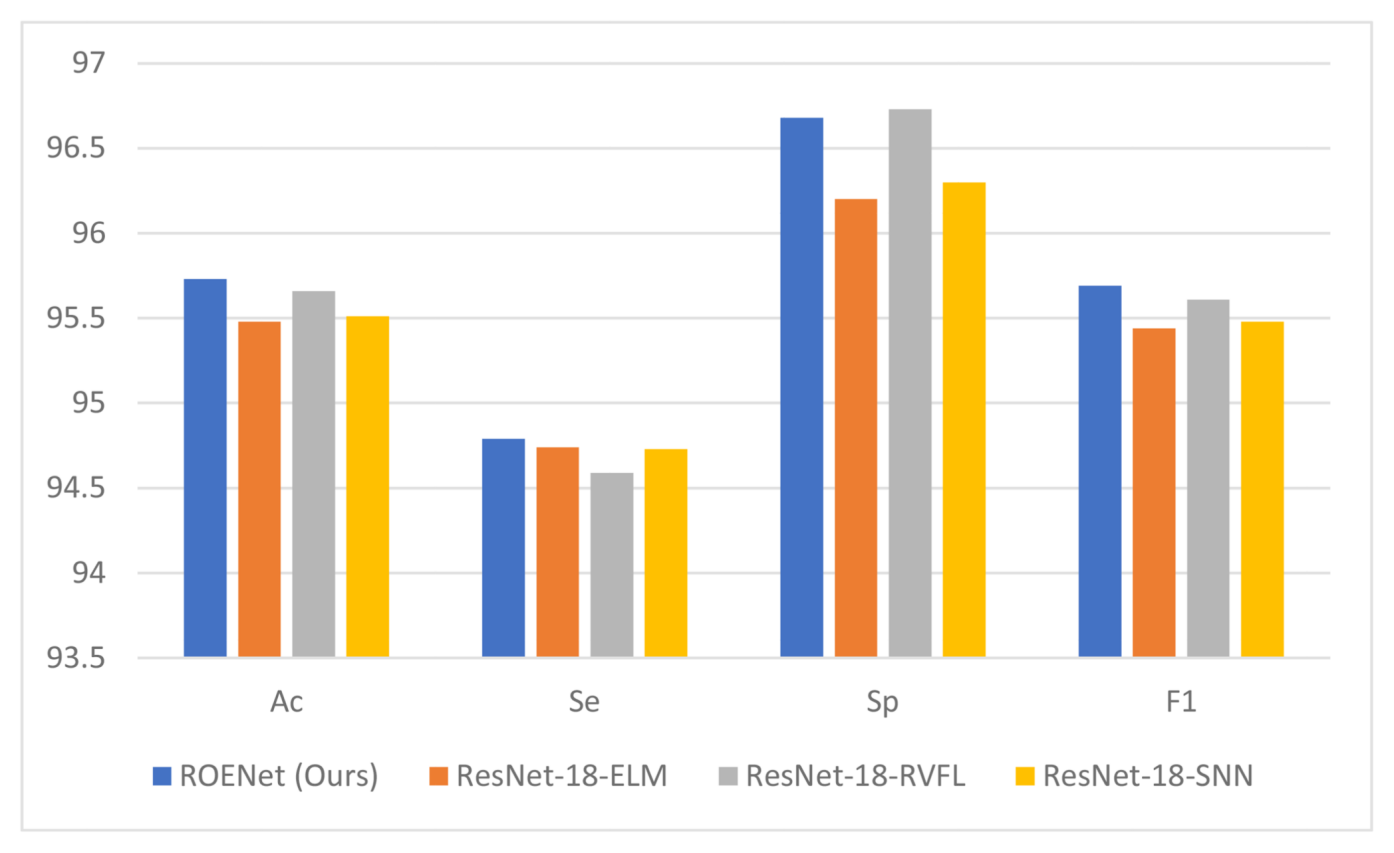
Effects of output ensemble.

In this paper, the results of ROENet are the ensemble outputs from three RNNs. To verify the superiority of the proposed ROENet, the proposed model is compared with three individual models. The classification performances of three individual models are provided in Table 4. For a clearer comparison, the comparison figure is presented in Figure 7. Compared with the other three individual networks, our proposed network achieves the best results in accuracy, sensitivity, and F1 score. Although it is not the best in specificity, it is only 0.05% lower than the best.

### 4.5 Comparison with the Fine-Tuned ResNet-18

**Figure 8:**
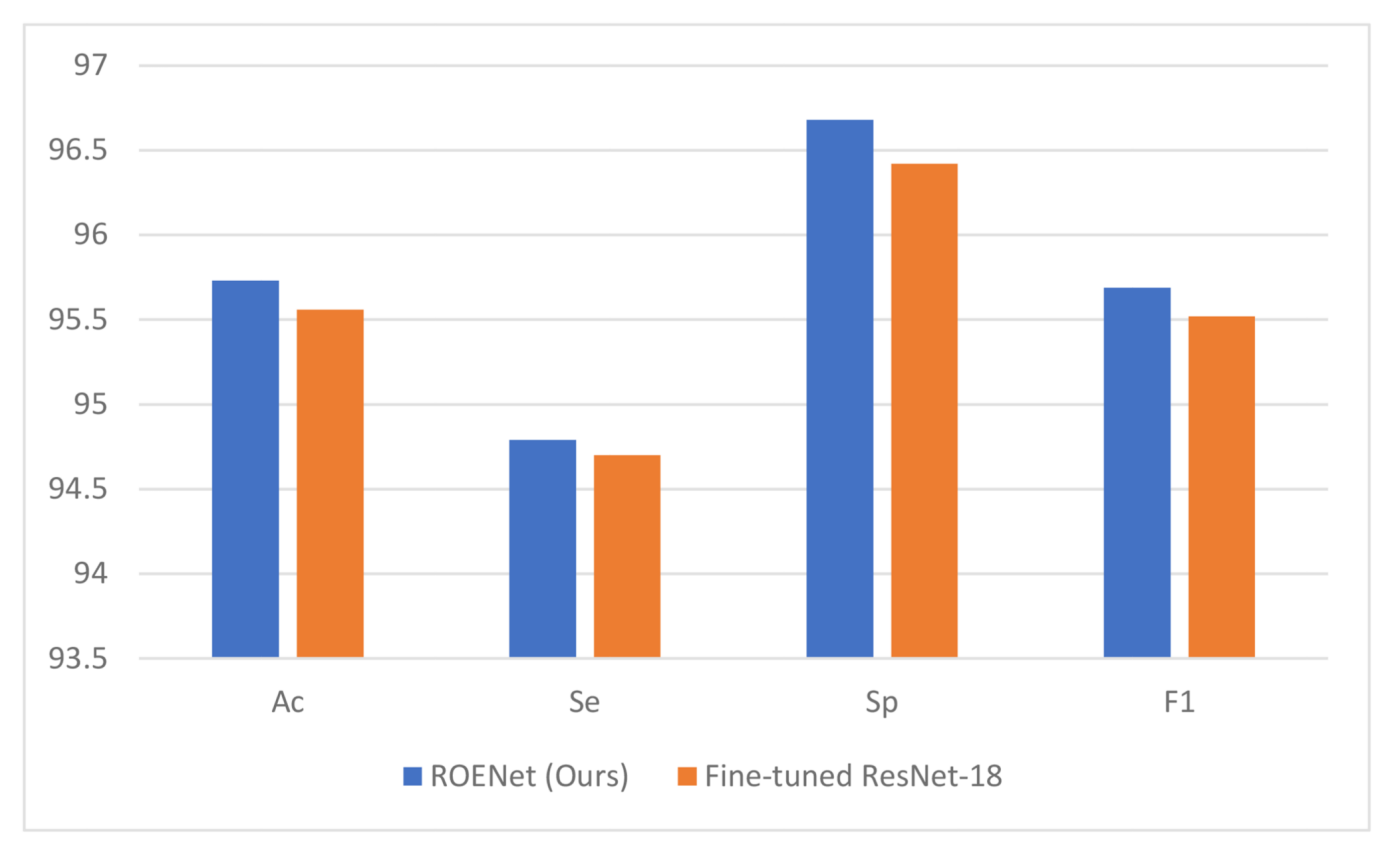
The comparison of the proposed model with the fine-tuned model.

We compare the ROENet with the fine-tuned ResNet-18. The results of the fine-tuned model are presented in Table 4. The comparison of the proposed model with the fine-tuned model is provided in Figure 8. Our proposed model achieves better results than the fine-tuned ResNet-18. This proves that our model is an effective tool to classify malaria parasite on the blood smear.

The parameters and layers of RNNs are less than those of ResNet-18. Because the dataset in this paper is not very large, RNN is unlikely to have the problem of overfitting. Therefore, the proposed ROENet overperforms the fine-tuned ResNet-18.

### 4.6 Comparison with Other State-of-the-Art Methods

**Figure 9:**
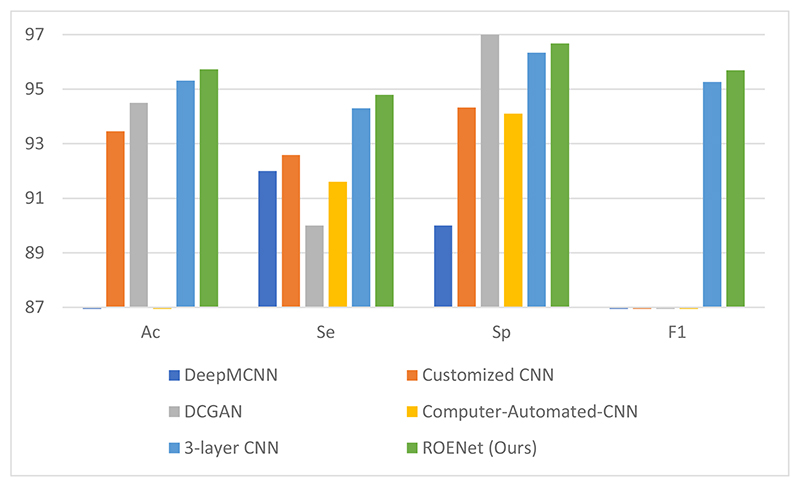
The comparison with other state-of-the-art methods.

ROENet is compared with other state-of-the-art (SOTA) methods, which are Deep-MCNN [1], Customized CNN [2], DCGAN [3], Computer-Automated CNN [4], and three-layer CNN [9], respectively. DCGAN, Computer-Automated CNN, and three-layer CNN used the same dataset as this paper. Other SOTA methods used different datasets. The comparison is provided in Table 5. The comparison figure is presented in Figure 9. From the table and figure, we can see that our model obtains the best results in comparison with other SOTA methods.

There are three reasons why our model can achieve better results than other SOTA methods. (i) ResNet-18 is the backbone of our model, which can accurately extract features. (ii) We use RNN as the classifier, which can avoid overfitting problems. (iii) The results of ROENet are the ensemble outputs from three RNNs, which can improve the classification performance.

## 5 Conclusions

This paper proposes a new method (ROENet) to classify malaria parasite on the blood smear automatically. The backbone of the ROENet is the pre-trained ResNet-18. The output nodes of the pretrained ResNet-18 are 1000. However, the output nodes are two in this paper. Therefore, we perform some modifications to the backbone. We use randomized neural networks (RNNs) as the classifier in our proposed model, because the structure of RNN is simpler than ResNet-18. Three RNNs are used in ROENet, which are random vector functional link (RVFL), extreme learning machine (ELM), and Schmidt neural network (SNN). To improve the performance of ROENet, the results of ROENet are the ensemble outputs from three RNNs. We evaluate the proposed ROENet by fivefold cross-validation. The specificity (Sp), F1 score (F1), sensitivity (Se), and accuracy (Ac) are 96.68 ± 3.81%, 95.69 ± 2.65%, 94.79 ± 3.71%, and 95.73 ± 2.63%, respectively. The proposed ROENet is compared with other SOTA methods and provides the best classification performance among these methods, which proves that our model is an effective tool to classify malaria parasite on the blood smear.

Even though our model obtains excellent classification performance, there are still some limitations. (i) The dataset is still small. (ii) We only tested on one public dataset.

In future work, we will collect more datasets to test our model and continue to improve the performance and robustness of our model to better classify malaria parasite on the blood smear. Furthermore, we will try other latest methods to classify malaria parasite on the blood smear, such as VIT.

## Figures and Tables

**Table 1:** Acronym and Full Explanation.

Acronym	Full Explanation
Ac	Accuracy
Avr	Average
BN	Batch normalization
CNN	Convolution neural network
ELM	Extreme learning machine
F1	F1 score
FC	Fully connected
ML	Machine learning
RVFL	Random vector functional link
RNNs	Randomized neural networks
Se	Sensitivity
SNN	Schmidt neural network
Sp	Specificity
Std	Standard deviation

**Table 2:** The pseudocode of ROENet.

Pseudocode of ROENet
Step 1 The backbone is the pre-trained ResNet-18.
Step 2: Some modifications are made to the backbone.
Step 2.1 FC1000, Softmax, and classification layer are removed.
Step 2.2 Some layers (FC128, ReLU, BN, FC2, softmax, and classification layer) are added.
Step 3: The malaria data set is divided into five groups of the same size and set *i* = 1.
Step 4: The *i*-th group is chosen as the test set, and the training set is formed by other groups.
Step 5: The modified ResNet-18 is fine-tuned.
Step 5.1: The training set is input.
Step 5.2: The label of the training set is the target.
Step 6: The output of the FC128 layer is the features.
Step 7: Three RNNs (Randomized Neural Networks) are used to replace the end of five layers of the fine-tuned backbone.
Step 8: The classifier of the ROENet is trained on the dataset.
Step 8.1: The features are the input.
Step 8.2: The labels of the training set are the target.
Step 9: The final results are the ensemble outputs from three RNNs.
Step 10: The results of the proposed ROENet are reported.
Step 11: Set *i* = *i* + 1, if *i* < 6, go to Step 4.
Step 12: Average test classification performance.

**Table 3:** The hyper-parameters of the ROENet.

Hyper-Parameters	Value
Max-epoch	4
Learning rate	10^−4^
Minibatch size	128
Number of the hidden nodes *u*	400

**Table 4:** The classification performance.

Methods	Fold	Ac	Se	Sp	F1
ROENet (Ours)	F 1	95.41	94.34	96.48	95.36
	F 2	95.59	95.14	96.04	95.57
	F 3	96.03	95.14	96.92	95.99
	F 4	96.06	94.99	97.13	96.02
	F 5	95.57	94.34	96.81	95.52
	Avr	95.73	94.79	96.68	95.69
	Std	±2.63	±3.71	±3.81	±2.65
AlexNet-OE	F 1	95.61	95.71	95.43	95.62
	F 2	95.63	96.01	95.25	95.64
	F 3	95.17	95.79	94.56	95.20
	F 4	94.97	95.46	94.48	94.99
	F 5	95.66	95.79	95.53	95.67
	Avr	95.41	95.75	95.05	95.42
	Std	±2.84	±1.77	±4.43	±2.77
ResNet50-OE	F 1	95.34	94.74	95.94	95.31
	F 2	94.99	94.56	95.43	94.97
	F 3	95.57	94.99	96.15	95.55
	F 4	95.41	94.99	95.83	95.39
	F 5	95.28	94.88	95.68	95.26
	Avr	95.32	94.83	95.81	95.30
	Std	±1.91	±1.64	±2.43	±1.90
ResNet-18-ELM	F 1	94.99	94.23	95.75	94.95
	F 2	95.17	94.66	95.68	95.15
	F 3	96.05	95.46	96.63	96.02
	F 4	95.65	94.74	96.55	95.61
	F 5	95.52	94.63	96.41	95.48
	Avr	95.48	94.74	96.20	95.44
	Std	±3.72	±3.99	±4.06	±3.72
ResNet-18-RVFL	F 1	95.10	93.72	96.48	95.03
	F 2	95.68	95.03	96.33	95.65
	F 3	95.95	95.21	96.70	95.92
	F 4	96.01	94.88	97.13	95.96
	F 5	95.56	94.09	97.02	95.49
	Avr	95.66	94.59	96.73	95.61
	Std	±3.26	±5.78	±3.06	±3.38
ResNet-18-SNN	F 1	95.37	94.48	96.26	95.33
	F 2	95.37	94.99	95.75	95.35
	F 3	95.83	94.92	96.73	95.79
	F 4	95.83	95.07	96.59	95.80
	F 5	95.17	94.19	96.15	95.13
	Avr	95.51	94.73	96.30	95.48
	Std	±2.68	±3.39	±3.45	±2.68
Fine-tuned ResNet-18	F 1	95.23	94.38	96.08	95.19
	F 2	95.44	94.81	96.08	95.42
	F 3	95.94	95.36	96.52	95.91
	F 4	95.83	94.85	96.81	95.79
	F 5	95.36	94.09	96.63	95.30
	Avr	95.56	94.70	96.42	95.52
	Std	±2.76	±4.35	±2.96	±2.80

OE means output ensemble.

**Table 5:** The comparison with other SOTA methods.

Methods	Ac	Se	SP	F1
DeepMCNN [1]	-	92.00	90.00	-
Customized CNN [2]	93.46	92.59	94.33	-
DCGAN [3]	94.50	90.00	99.00	-
Computer-Automated-CNN [4]	-	91.60	94.10	-
3-layer CNN [9]	95.32	94.30	96.34	95.27
ROENet (Ours)	95.73	94.79	96.68	95.69

## Data Availability

Publicly available datasets were analyzed in this study. These data can be found here: https://lhncbc.nlm.nih.gov/LHC-downloads/dataset.html (accessed on 16 January 2022).
